# A Universal Approach to Molecular Identification of Rumen Fluke Species Across Hosts, Continents, and Sample Types

**DOI:** 10.3389/fvets.2020.605259

**Published:** 2021-03-04

**Authors:** Gillian Mitchell, Ruth N. Zadoks, Philip J. Skuce

**Affiliations:** ^1^Moredun Research Institute, Pentlands Science Park, Penicuik, United Kingdom; ^2^Sydney School of Veterinary Science, Faculty of Science, University of Sydney, Camden, NSW, Australia

**Keywords:** rumen fluke, paramphistome, livestock, wildlife, ITS2 sequencing

## Abstract

Rumen fluke are parasitic trematodes that affect domestic and wild ruminants across a wide range of countries and habitats. There are 6 major genera of rumen fluke and over 70 recognized species. Accurate species identification is important to investigate the epidemiology, pathophysiology and economic impact of rumen fluke species but paramphistomes are morphologically plastic, which has resulted in numerous instances of misclassification. Here, we present a universal approach to molecular identification of rumen fluke species, including different life-cycle stages (eggs, juvenile and mature fluke) and sample preservation methods (fresh, ethanol- or formalin-fixed, and paraffin wax-embedded). Among 387 specimens from 173 animals belonging to 10 host species and originating from 14 countries on 5 continents, 10 rumen fluke species were identified based on ITS-2 intergenic spacer sequencing, including members of the genera *Calicophoron, Cotylophoron, Fischeroedius, Gastrothylax, Orthocoelium*, and *Paramphistomum*. Pairwise comparison of ITS-2 sequences from this study and GenBank showed >98.5% homology for 80% of intra-species comparisons and <98.5% homology for 97% of inter-species comparisons, suggesting that some sequence data may have been entered into public repositories with incorrect species attribution based on morphological analysis. We propose that ITS-2 sequencing could be used as a universal tool for rumen fluke identification across host and parasite species from diverse technical and geographical origins and form the basis of an international reference database for accurate species identification.

## Introduction

Rumen fluke, or paramphistomes, are parasitic trematodes that reside in the digestive tracts of ruminants, including livestock such as cattle (1, 2), sheep (3, 4), goats (5, 6), and farmed deer (7); camelids (8) and wildlife, such as antelope (9–11), buffalo (12), and various species of wild deer (12, 13).

The first published information on rumen fluke emerged in 1790, describing adult fluke within the rumen of red deer in Europe (14). Multiple changes were subsequently made to the nomenclature until Fischoeder created the genus *Paramphistomum* in 1903. There is a lack of full agreement or consistency around nomenclature in the literature, with the names rumen fluke and stomach fluke or paramphistome and amphistome largely used interchangeably (15). They mainly belong to six different families: Paramphistomidae, Gastrodiscidae, Gastrothylacidae, Olveriidae, Balanorchiidae, and Stephanopharyngidae; all in the Superfamily Paramphistomoidea, Stiles and Goldberger, 1910 (16); under which, more than 70 species have been described (17).

Paramphistomes have a worldwide distribution, with different species endemic in specific areas and host species. In some countries, multiple species of rumen fluke are present, sometimes existing as co-infections in a single host animal, e.g., in India (18) and Japan (19). *Calicophoron daubneyi* (formerly *Paramphistomum daubneyi*) is the dominant species in Europe, e.g., in Spain (2), Italy (20), the United Kingdom and Ireland (4, 21, 22). Some species are found across multiple host species in geographically distant areas, e.g., *Paramphistomum leydeni* has been found in goats in China (6), cattle in the Netherlands (23) and Argentina (3), sheep in Ireland (24), and reindeer in Finland (25).

Adult rumen fluke appear to pose little or no significant concerns for animal health and productivity (26). Heavy infestations of immature rumen fluke feeding on the intestinal lining, however, can cause disease, known as larval paramphistomosis (27). In India, mortality rates due to immature paramphistomes may reach 80–90% in domestic ruminants (28), which might be associated with differences in rumen fluke species or burden, or the nutritional, health, immune, and genetic status of the host. There is growing concern that rumen fluke prevalence is increasing significantly in Europe (29, 30). For example, in Ireland, prevalence of paramphistomes, based on detection of rumen fluke eggs in feces, increased from 3% in 2009 to around 28% in 2013 in cattle (21). Concurrently, Toolan et al. (2015) reported a rise in prevalence of rumen fluke eggs detected at local Veterinary Investigation Centers between 2010 and 2013, from 12.4 to 22% in sheep. Increased rumen fluke prevalence may lead to an increase in cases of acute larval paramphistomosis (31). Indeed, there is evidence of the disease in cattle in Ireland (32), calves in England (33); and even mortality in adult sheep in Scotland (34).

Diagnosis of rumen fluke infection can be based on inspection of the reticulo-rumen for presence of adult flukes at slaughter (7, 22), on detection of rumen fluke eggs in feces (4, 21), or on detection of immature paramphistomes in excreta or at post mortem (35). Rumen fluke eggs are very similar morphologically to eggs from the liver fluke, *Fasciola hepatica*, which could lead to misdiagnosis of infection status and treatment response (4). In addition, rumen fluke are a “plastic” group morphologically, with variability in size and pigmentation. For example, in our previous work, rumen fluke from sheep appeared smaller than those from cattle, even though they proved to belong to the same species. Conversely, the finding of *C. daubneyi* and *P. leydeni* in cattle in the Netherlands was a surprise, both because *P. leydeni* is relatively unknown, but also because the two species could not be differentiated morphologically (23).

Accurate species identification is important for understanding of the rumen fluke life-cycle and control options. Until the mid-2010s, it was assumed that the predominant rumen fluke in the British Isles belonged to the species, *Paramphistomum cervi*, which has an aquatic planorbid snail as intermediate host (27). Using molecular identification, Gordon et al. (2013) identified that rumen fluke infecting sheep in Scotland were, in fact, *C. daubneyi*, a finding since confirmed by others for sheep and cattle across the whole of the UK and Ireland (21, 22). *C. daubneyi* does not have an aquatic snail as intermediate host, rather, it uses the same mud snail, *Galba truncatula*, as the liver fluke (36, 37). This represents a radical change in our understanding of rumen fluke epidemiology, which may include ecological competition for snail hosts and encystment habitats between rumen fluke and liver fluke. It may also be important in understanding the emergence of rumen fluke, as this may be linked to changes in climatic conditions that favor survival of intermediate mud snail host species (38) or cercarial life stages on pasture (39).

Species identification and taxonomy of rumen fluke were initially based on a system introduced by Näsmark (40), with identification by morphological and histological methods. More recently, this approach has been refined through light microscopy (1, 25, 41) and detailed scanning electron microscopy (25, 42, 43). These methods are, however, laborious, technically demanding, low throughput and time-consuming, and have resulted in several instances of misidentification and generation of synonyms (44, 45). Those problems can be overcome with molecular methods such as the polymerase chain reaction (PCR), to amplify specific regions of rumen fluke DNA, followed by sequencing of the amplified region (17). Commonly used DNA markers include nuclear ribosomal genes 28S and 18S (46) and their related spacers, and mitochondrial DNA (mtDNA) c oxidase subunit I (Cox1) genes (21, 47). The most frequently used and versatile genetic marker for phylogenetic analysis has been the internal transcribed spacer (ITS-2) region of ribosomal DNA (rDNA), which has been used successfully in Europe (20, 48, 49), South America (3), and Asia (17, 18, 50).

As laboratory methodology and reagents for DNA extraction and amplification from various sample types become more sophisticated, and awareness of the importance and emergence of rumen fluke grows, there is an opportunity to conduct both prospective and retrospective studies of rumen fluke epidemiology with accurate species identification using archived and freshly collected material. The aim of our study was to establish a universal approach to rumen fluke identification across fluke species, host species, continents and specimen types. In addition to identification from adult and juvenile rumen fluke obtained at post mortem and identification using DNA extracted from rumen fluke eggs, the use of DNA from ethanol-, formalin- and paraffin wax-preserved specimens was examined to allow for use of historical archived specimen collections.

## Materials and Methods

### Parasite Material

Samples were collected by farmers, veterinarians and scientists across the world (see Acknowledgments for list of those who submitted) for diagnostic or research purposes. These were made available for this study to ascertain the suitability of our methodology across countries, host species, parasite species and sample types, and to generate preliminary data on the geographic and host-species distribution of paramphistome species. A total of 387 specimens were processed, including at least 2 or 3 individual specimens extracted from 173 host animals native to: Australia (2), Belgium (2), Cuba (6), Dominican Republic (1), India (1), Ireland (46), Italy (3), the Netherlands (17), New Caledonia (51), Slovakia (2), St. Kitts (7), Tanzania (2), the UK (33), and the USA (1).

Adult fluke (*n* = 344) were obtained from the reticulo-rumen and juveniles (*n* = 20) from the intestines of naturally infected ruminants and camelids at slaughter or post-mortem. The animals examined comprised of: alpaca (2), bison (1), cattle (112), goat (3), llama (2), red deer (2), reindeer (1), rusa deer (13), sheep (36), and water buffalo (1). In addition, a limited number of formalin-fixed (*n* = 4, i.e., two adult fluke each from two cows in Northern Ireland) and wax-embedded (*n* = 3 individual flukes, collected from one cow in England) samples were included. Those samples had originally been prepared for histopathological examination, and were used to provide proof of concept for the applicability of the proposed approach to archived sample material. Rumen fluke eggs (*n* = 23) were either collected at the Moredun Research Institute (*n* = 6 from two individual fecal samples and *n* = 6 from one pooled fecal sample) using published methods (52), or received extracted and in ethanol (*n* = 11 from four different host animals).

### Genomic DNA Extraction

DNA was extracted from individual adult flukes or pools of immatures of ~25 mg. Samples stored in formalin were washed three times in 1X phosphate buffered saline (PBS) prior to DNA extraction. For eggs stored in ethanol, 200 μl aliquots were left to sediment for 3 min, before siphoning off the excess ethanol and rinsing three times in 1X PBS to ensure all eggs were free of sediment, and repeating sedimentation. DNA extraction was performed using Qiagen DNEasy Blood and Tissue kit (QIAGEN, Germany) as specified by the manufacturer. The lysis step involving Proteinase K was performed overnight for ethanol-fixed as well as formalin-fixed samples. For paraffin wax-fixed samples, sections of host and parasite tissue were cut from wax blocks provided by APHA, Lasswade. Initial attempts to extract DNA from wax blocks using the Qiagen kit were not successful. Therefore, DNA from paraffin wax-fixed samples was extracted using Ambion RecoverAll Total Nucleic Acid Isolation Kit (Life Technologies, USA) as per the manufacturer's protocol.

### Polymerase Chain Reaction

The ITS-2 rDNA, plus partial flanking 5.8S and 28S regions were amplified using the generic trematode primers, ITS-2Trem (Table 1) (19, 20). PCR was conducted using a reaction volume of 25 μl containing 10x Buffer (Invitrogen, USA), 12.5 pmol of each primer (Eurofins, Germany), 0.2 mM of each dNTP (Invitrogen, USA), 2 mM MgCl_2_ (Invitrogen, USA), 2.5U platinum *Taq* polymerase (Invitrogen, USA) and 1 μl of template DNA. The PCR was performed in a GeneAMP PCR system 2720 thermal cycler (Applied Biosystems, USA) under the following conditions: 95°C for 10 min; 35 cycles of 94°C for 1 min; 53°C for 1.5 min; 72°C for 1 min; and 72°C 10 min. 5 μl of the PCR product was viewed in 1.2% agarose gel prepared in Tris-acetate-EDTA (TAE) buffer with GelRed (Biotium, USA) and visualized on an Alphamagel Imaging System (Alpha Innotech, USA). PCR products of the appropriate size (~500 bp) were purified using QIAquick PCR Purification Kit (QIAGEN, Germany) as specified by the manufacturer. Products were eluted in 30 μl Elution Buffer.

**Table 1:** PCR primer names, sequences, and product size.

**Primer set (Source)**	**Primer name**	**Sequence (5^**′**^ → 3^**′**^)**	**Product size (bp)**
ITS-2 Trematode (19)	ITS-2Trem F	TGTGTCGATGAAGAGCGCAG	428–500
	ITS-2Trem R	TGGTTAGTTTCTTTTCCTCCGC	
ITS-2 Shorter (In-house)	ITS-2Short F	GTAACAGAACACCACAGTAGGT	<200
	ITS-2Short R	CCGGACACAACCGCGTCTTGCTGG	
28S Digenea (46)	Dig28S F	AAGCATATCACTAAGCGG	~900
	Dig28S R	GCTATCCTGAGGGAAACTTCG	

For rumen fluke DNA that appeared to be damaged as a result of prior processing, e.g., formalin fixation, (see Results), primers were designed to amplify a smaller ITS-2 fragment within the larger ITS-2 region of <200 bp (ITS-2Short, Table 1). The reagent volumes were as previously described, but with a total reaction volume of 50 μl. PCR conditions were: 95°C for 10 min; 40 cycles of 95°C for 30 s; 58°C for 30 s; 72°C for 1 min; and 72°C 10 min. 20 μl of PCR product was run on a 2% agarose gel, the gel was inspected on an ultraviolet transilluminator (Thermo Scientific, USA), bands of the correct amplicon length were excised, and DNA extracted using a QIAquick Gel Extraction Kit (QIAGEN, Germany), according to the manufacturer's instructions.

Where species identity based on ITS-2 sequence data was ambiguous, e.g., because a perfect match was not found in online reference databases, the large subunit ribosomal DNA 28S was also examined. Amplification of the 900 bp 28S rRNA fragment was achieved by use of Dig28S primers (Table 1) (46). Master Mix and PCR parameters were as described above for amplification of ITS-2, but with annealing temperature 56°C.

The specificity of primers for rumen fluke species was tested against a panel of control trematode and gastrointestinal nematode DNA extracts originating from bovine or ovine hosts (except for the free-living planarian, *Arthurdendyus triangulatus*; and the fish eye fluke, *Diplostomum spathaceum*) (Table 2). Apart from *A. triangulatus* (0.25 g section used) and *Fasciola gigantica* (extracted from a crude lysate), all samples were prepared from single adult worms using the method described above. A positive control of 10 ng/μl *C. daubneyi* DNA and a negative control of nuclease-free (NF) H_2_O were included in each PCR assay.

**Table 2:** Nematode and trematode species used as positive (*Calicophoron daubneyi*) or negative (all other species) controls in PCR assays for detection and species identification of rumen fluke.

**Phylum**	**Species**	**Location**	**Host species**
Platyhelminth	*Arthurdendyus triangulatus*	Scotland; Edinburgh	N/A (garden soil)
	*Calicophoron daubneyi*	Scotland; Glasgow	Cattle
	*Dicrocoelium dendriticum*	Scotland; Coll	Sheep
	*Diplostomum spathaceum*	Scotland; Loch Etive	Rainbow trout
	*Fasciola gigantica*	India; Tamil Nadu	Unknown
	*Fasciola hepatica*	Scotland; Edinburgh	Sheep
Nematode	*Chabertia ovina*	Scotland; Edinburgh	Sheep
	*Cooperia curticei*	Scotland; Edinburgh	Sheep
	*Haemonchus contortus*	Scotland; Edinburgh	Sheep
	*Nematodirus battus*	Scotland; Edinburgh	Sheep
	*Teladorsagia circumcincta*	Scotland; Edinburgh	Sheep
	*Trichostrongylus axei*	Scotland; Edinburgh	Sheep
	*Trichostrongylus colubriformis*	Scotland; Edinburgh	Sheep
	*Trichostrongylus vitrinus*	Scotland; Edinburgh	Sheep

### Sequence and Phylogenetic Analysis

Purified PCR products were analyzed on a NanoDrop 1000 Spectrophotometer (Thermo Scientific, USA) to determine quantity and purity of DNA, before sending to Eurofins MWG Operon (Germany) at a concentration of 5 ng/μl for direct nucleotide sequencing. From each infected animal, at least two individual flukes were sequenced. The chromatogram files were aligned using Lasergene 10 core suite software SeqMan Pro (DNASTAR, USA) to assess the quality of the sequences and, where necessary, assemble the full fragment length. The final sequences were compared to reference sequences in GenBank using BLASTn at the European Bioinformatics Institute website (http://www.ebi.ac.uk/).

For phylogenetic analysis, sequences were aligned by MUSLE using Alignment Explorer in MEGA6 (53). For each assembled sequence, a nucleotide query was conducted using NCBI BLAST and the closest matching sequences downloaded in full. Duplicate reference sequences, sequences originating from “unidentified paramphistome” or unverified with regards to source in GenBank, and sequences with <98% percentage identity or <98% query cover compared to any of the sequences generated in the current study were manually removed. The remaining reference sequences, together with any control sequences previously generated in-house using relevant primers, were added to the MEGA6 alignment. All sequences were manually cropped to equal lengths, i.e., 364 and 831 nt for ITS-2 and 28S, respectively. A Neighbor-Joining phylogenetic tree was constructed in Tree Explorer to assess the level of heterogeneity. The evolutionary distances were estimated using the Maximum Composite Likelihood method in MEGA, which accounts for multiple hits, heterogeneity of substitutions patterns between nucleotides, and inequality of nucleotide frequencies (53). Support for the tree topology was assessed by the bootstrap method with 1,000 pseudoreplicates. For the 10 species identified in the current study, the Pairwise Distance function in MEGA6 was utilized to calculate % homology for pairs that had been assigned the same species names (intra-specific) or different species names (inter-specific). All available sequences available on GenBank (155) were used (trimmed to 299 bp segment to allow for inclusion of sequences that were shorter than those generated in the current study), together with unique sequences generated in this study, i.e., one for the majority of species, and two sequences for three species showing intraspecies heterogeneity; Supplementary Table 1).

## Results

The generic ITS-2 Trem primers were successful in amplifying DNA extracted from whole adult rumen fluke (*Calicophoron calicophorum, Calicophoron microbothrium, Cotylophoron cotylophorum, C. daubneyi, Fischeroedius elongatus, Gastrothylax crumenifer, Orthocoelium streptocoelium, P. cervi*, and *P. leydeni*); pools of juvenile rumen fluke (*C. daubneyi*); pools of rumen fluke eggs (*C. daubneyi, Calicophoron microbothrioides*, and *P. leydeni*); adult liver fluke (*F. hepatica*) and lancet fluke (*D. dendriticum*) and from crude liver fluke lysates (*F. gigantica*). In most cases the entire ITS-2 region (~282 bp) plus partial flanking 5.8s and 28S sequence was amplified as anticipated. Multiple weak bands were obtained for the lancet fluke, and an additional platyhelminth species, the New Zealand flatworm, *A. triangulatus* (Class: Turbellaria) (Supplementary Figure 1). No amplification was seen in any of the gastrointestinal nematode species tested (data not shown).

DNA extracts from paraffin wax-embedded samples and those stored in formalin for over a year (extracted from *C. daubneyi*, UK) did not yield sufficient quality PCR product for sequencing when using the generic ITS-2Trem primers. In these instances, the ITS-2Short primers designed to amplify a smaller ITS-2 section were successful in producing 171–209 bp sized fragments, omitting the first ~200 bp of the larger ITS-2 fragment. This section had a high enough degree of divergence in the trematodes examined in this study to discriminate between species, the lowest nucleotide difference being 3 base differences (between *C. microbothrium* and *C. calicophorum*).

**Figure 1:**
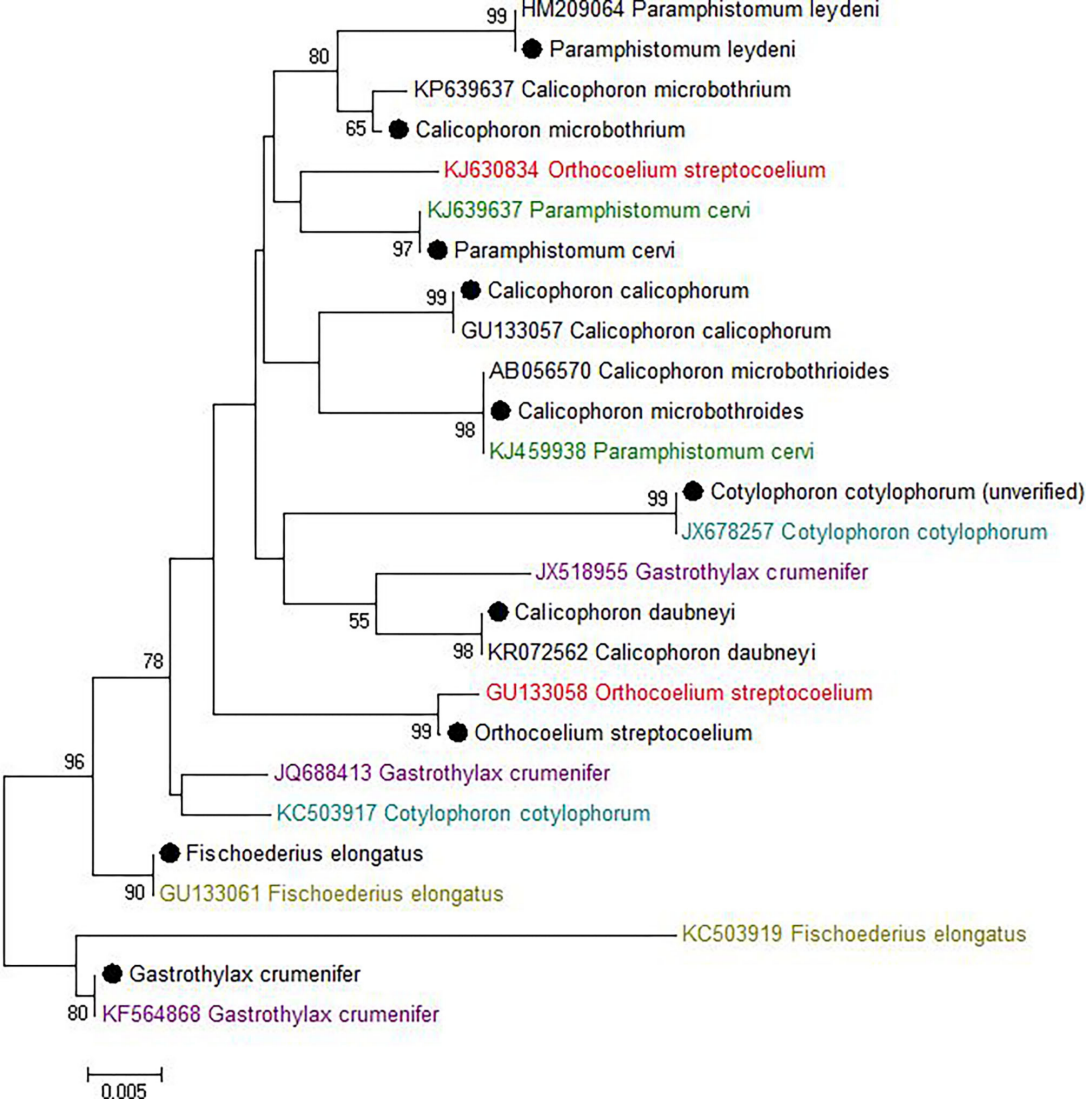
Neighbor-joining tree of unique ITS-2 sequences (364 nt) generated in this study (•) and those available in GenBank (Accession number preceding species name). The percentage of replicate trees in which the associated taxa clustered together in the bootstrap test (1,000 replicates) is shown next to the branches. The evolutionary distances were computed using the Maximum Composite Likelihood method and are displayed as number of base substitutions per site. Bootstrap values of <50% are not shown.

**Figure 2:**
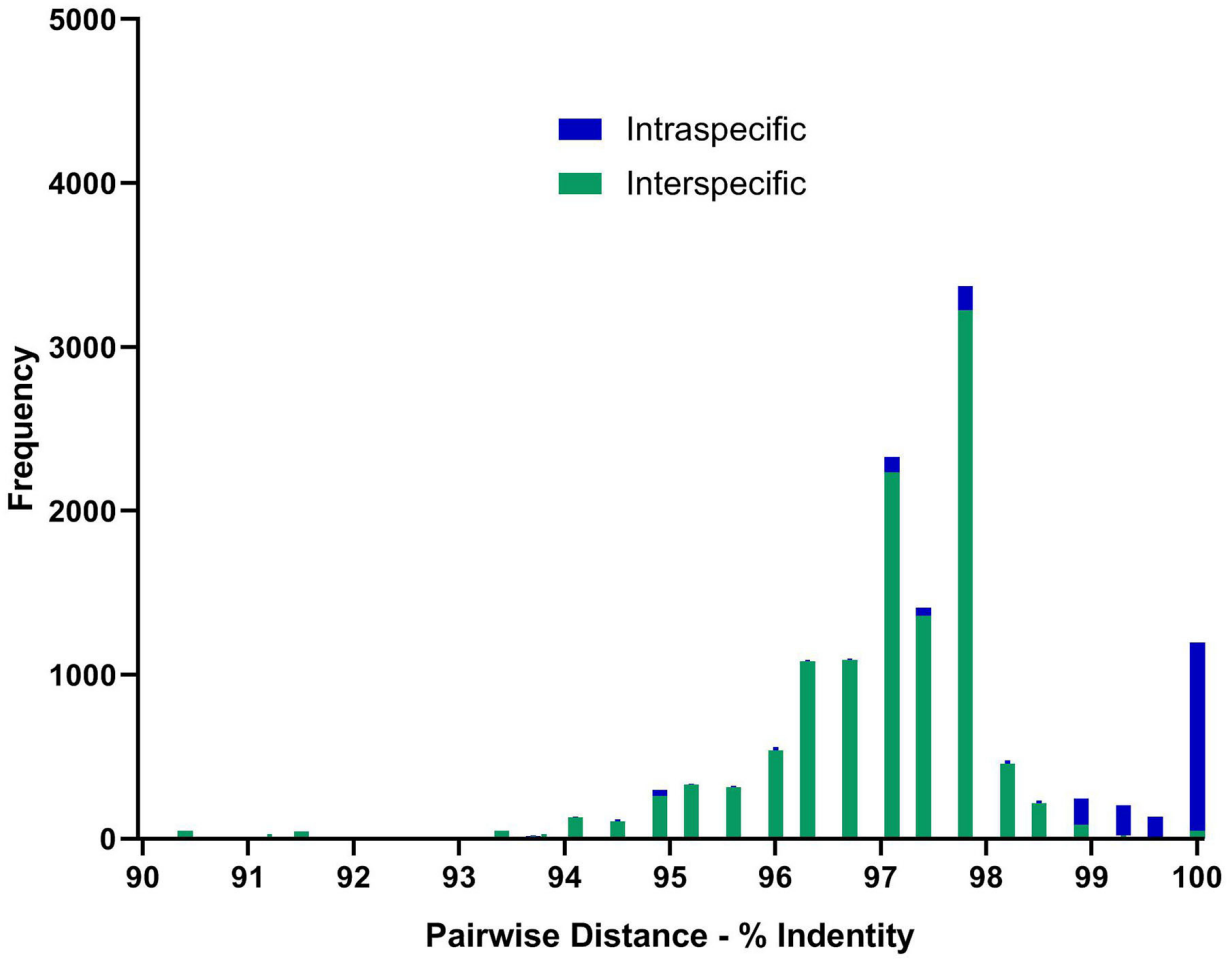
Distribution of pair-wise sequence identity between all available ITS-2 GenBank (*n* = 155) sequences and unique in-house generated sequences (*n* = 13) for the 10 paramphistome species found in this study. Green bars showing nucleotide differences between species and blue within species. Sequences were aligned using MEGA6 and trimmed to the same length of 299 bp before determining the number of base differences per site between sequences using the Pairwise Distance computation function. Pairs showing <90% homogeneity were omitted, but can be seen in Supplementary Figure 2 and Supplementary Table 2.

Most sequences derived from our collection showed 99.6–100% identity to the species matched most closely in GenBank (Figure 1). By contrast, GenBank-derived sequences clustered in multiple clades for *O. streptocoelium, P. cervi, C. cotylophorum*, and *F. elongatus* (two clades each) and *G. crumenifer* (three clades) with bootstrap support of 90–99% for nodes separating clades (Figure 1). Based on pair-wise comparison, intraspecific homology was >98.5% for 80% of sequence pairs, compared to <98.5% for 97% of interspecific comparisons, with a small number of intra-specific comparisons falling in the distribution observed for inter-specific comparisons, or vice versa (Figure 2).

For one species, *C. cotylophorum*, which was uniquely associated with rumen fluke from St. Kitts in our study, very few ITS2 sequences were available for comparison. The ITS2 sequences (*n* = 16) from St. Kitts were 100% identical to each other and to *C. cotylophorum* from *Bos indicus* (zebu) in Tripura, India [JX678257 (46) but distinct from the second sequence in GenBank reported to originate from *C. cotylophorum*, also isolated from Indian zebu - KC503917. To obtain greater certainty about the identity of specimens from St. Kitts, 28S RNA gene sequencing was also used. Identification as *C. cotylophorum* was supported by 100% identity to an 803 bp segment of the 28S rRNA gene [JX678278, (46)], which originates from the same study as the closest ITS-2 match (Supplementary Figure 3).

With the exception of *C. cotylophorum*, every rumen fluke species that was identified more than once was found in more than one host species, often including livestock and wildlife (e.g., *C. calicophorum, F. elongatus, O. streptocoelium*, and *P. leydeni*) or livestock and camelids (*C. daubneyi*) (Table 3). Multiple rumen fluke species per host species were identified in livestock (cattle, sheep, goats) and Rusa deer, whereas only a single rumen fluke species was identified in most camelids and wildlife species, possibly reflecting the number of samples included in the study. The three most common rumen fluke species were identified in more than one country (Table 3), as was *P. leydeni*, whereby the countries could be geographically clustered (e.g., for *C. daubneyi* and *P. leydeni* in Europe), or distant (e.g., for *C. microbothrioides* in the USA and Dominican Republic, or *C. microbothrium* in Cuba and Tanzania). Per country, one or more rumen fluke species could be identified, although this was largely an artifact of the study design, which was opportunistic and aimed at maximizing species diversity to allow rigorous assessment of the typing methodology across rumen fluke species from different host species and geographic origins.

**Table 3:** Paramphistome species identified in multiple host species across multiple geographic locations.

**Rumen fluke species**	**Host species**	**Country**													**UK**				**Total**
		**Australia**	**Belgium**	**Cuba**	**Dom.Rep**.	**India**	**Italy**	**Netherlands**	**New Cale**.	**ROI**	**Slovakia**	**St. Kitts**	**Tanzania**	**USA**	**England**	**NI**	**Scotland**	**Wales**	
*Calicophoron calicophorum*	Cattle	**2**	**-**	**-**	**-**	**-**	**-**	**-**	**32**	**-**	**-**	**-**	**-**	**-**	**-**	**-**	**-**	**-**	**34**
	Rusa deer	**-**	**-**	**-**	**-**	**-**	**-**	**-**	**6**	**-**	**-**	**-**	**-**	**-**	**-**	**-**	**-**	**-**	**6**
	Goat	**-**	**-**	**-**	**-**	**-**	**-**	**-**	**1**	**-**	**-**	**-**	**-**	**-**	**-**	**-**	**-**	**-**	**1**
*Cotylophoron cotylophorum*	Cattle	**-**	**-**	**-**	**-**	**-**	**-**	**-**	**-**	**-**	**-**	**7**	**-**	**-**	**-**	**-**	**-**	**-**	**7**
*Calicophoron daubneyi*	Alpaca	**-**	**-**	**-**	**-**	**-**	**-**	**-**	**-**	**-**	**-**	**-**	**-**	**-**	**2**	**-**	**-**	**-**	**2**
	Cattle	**-**	**2**	**-**	**-**	**-**	**-**	**3**	**-**	***34***	**-**	**-**	**-**	**-**	***7***	**1**	**10**	**1**	**58**
	Llama	**-**	**-**	**-**	**-**	**-**	**-**	**-**	**-**	**-**	**-**	**-**	**-**	**-**	2	**-**	**-**	**-**	**2**
	Sheep	**-**	**-**	**-**	**-**	**-**	**3**	12	**-**	***11***	**-**	**-**	**-**	**-**	**2**	**-**	***7***	**-**	**35**
*Calicophoron microbothrioides*	Cattle	**-**	**-**	**-**	**1**	**-**	**-**	**-**	**-**	**-**	**-**	**-**	**-**	**-**	**-**	**-**	**-**	**-**	**1**
	Bison	**-**	**-**	**-**	**-**	**-**	**-**	**-**	**-**	**-**	**-**	**-**	**-**	1	**-**	**-**	**-**	**-**	**1**
*Calicophoron microbothrium*	Cattle	**-**	**-**	**6**	**-**	**-**	**-**	**-**	**-**	**-**	**-**	**-**	**-**	**-**	**-**	**-**	**-**	**-**	**6**
	Goat	**-**	**-**	**-**	**-**	**-**	**-**	**-**	**-**	**-**	**-**	**-**	**2**	**-**	**-**	**-**	**-**	**-**	**2**
*Fischoederius elongatus*	Cattle	**-**	**-**	**-**	**-**	**-**	**-**	**-**	**3**	**-**	**-**	**-**	**-**	**-**	**-**	**-**	**-**	**-**	**3**
	Rusa deer	**-**	**-**	**-**	**-**	**-**	**-**	**-**	**4**	**-**	**-**	**-**	**-**	**-**	**-**	**-**	**-**	**-**	**4**
*Gastrothylax crumenifer*	Water buffalo	**-**	**-**	**-**	**-**	**1**	**-**	**-**	**-**	**-**	**-**	**-**	**-**	**-**	**-**	**-**	**-**	**-**	**1**
*Orthocoelium streptocoelium*	Cattle	**-**	**-**	**-**	**-**	**-**	**-**	**-**	**2**	**-**	**-**	**-**	**-**	**-**	**-**	**-**	**-**	**-**	**2**
	Rusa deer	**-**	**-**	**-**	**-**	**-**	**-**	**-**	**3**	**-**	**-**	**-**	**-**	**-**	**-**	**-**	**-**	**-**	**3**
*Paramphistomum cervi*	Red deer	**-**	**-**	**-**	**-**	**-**	**-**	**-**	**-**	**-**	**1**	**-**	**-**	**-**	**-**	**-**	**-**	**-**	**1**
*Paramphistomum leydeni*	Cattle	**-**	**-**	**-**	**-**	**-**	**-**	**1**	**-**	**-**	**-**	**-**	**-**	**-**	**-**	**-**	**-**	**-**	**1**
	Sheep	**-**	**-**	**-**	**-**	**-**	**-**	**1**	**-**	**-**	**-**	**-**	**-**	**-**	**-**	**-**	**-**	**-**	**1**
	Red deer	**-**	**-**	**-**	**-**	**-**	**-**	**-**	**-**	**-**	**1**	**-**	**-**	**-**	**-**	**-**	**-**	**-**	**1**
	Reindeer	**-**	**-**		**-**	**-**	**-**	**-**	**-**	**-**	**-**	**-**	**-**	**-**	1	**-**	**-**	**-**	**1**
**Total**		**2**	**2**	**6**	**1**	**1**	**3**	**17**	**51**	**45**	**2**	**7**	**2**	**1**	**14**	**1**	**17**	**1**	**173**

*Dom.Rep, Dominican Republic; New Cale, New Caledonia; ROI, Republic of Ireland; NI, Northern Ireland. DNA was extracted from adult worms (shown as bold font), juveniles (shown as italics) or eggs (shown with underscore), or a from multiple sources as indicated by the combined formatting*.

## Discussion

Accurate identification of rumen fluke species is important for understanding of disease epidemiology and control options. Examples include studies of spread of infectious agents through animal movements (51), origins and biosecurity risks posed by non-indigenous species of parasitological importance (7) and recognition of relevant intermediate host snail species (4). Pathology and severity of paramphistomosis may also be affected by the rumen fluke species (21), although relatively little is known about this, potentially due in part to difficulties with species identification.

Historically, morphological identification has been used, which has led to some taxonomic confusion and errors. For example, Willmott (54) morphologically identified two rumen fluke species, *Paramphistomum hiberniae* and *Paramphistomum scotiae*, found in Irish and Scottish cattle, respectively, slaughtered at a Glasgow abattoir. Willmott suggested that *P. hiberniae* was present in the Netherlands, too, and suggested samples from France, marked as *P. cervi*, were likely to be *P. microbothrium* (since reclassified as *C. microbothrium*). Subsequently, Odening (44) reviewed this histologically, identifying *P. leydeni, P. scotiae* and *P. hiberniae* as later synonyms of *P. cervi*. Since then, and as exemplified in this study, *C. daubneyi* has been the only paramphistome species to be identified within Great Britain. It is unclear whether both species previously co-existed or whether one of them was previously misidentified (4). The distinction is of major relevance for rumen fluke control, because *P. cervi* uses aquatic planorbid snails as intermediate hosts whereas *C. daubneyi* uses mud snails. Incorrect classification of rumen fluke species could result in misdirected and hence ineffective control efforts.

While histological examination appears to be more reliable than morphological identification (41), both are still flawed in that variations within species can be noted due to a number of factors including host species, paramphistome age, fixation status and plane of section during slide preparation (17). To resolve these issues, molecular characterization is increasingly being used, especially ITS-2 sequencing. To amplify the ITS-2 region of the rRNA gene of paramphistomes, a range of different primers has been used for adult trematode specimens (3, 20, 49, 55), larvae (55), and eggs (19). Most studies focused on a limited number of countries, host species or sample types. Here, we demonstrate the utility of a universal set of ITS-2 primers across adult rumen fluke, juvenile rumen fluke, and fluke eggs from multiple hosts and locations in detection and identification of 10 rumen fluke species belonging to multiple genera. A suitable level of interspecific variation required for species identification existed within the ITS-2 region, although amplification of the full ITS-2 region was not always feasible where DNA was fragmented as a result of degradation through storage or fixation. Others have reported similar difficulties in the amplification of this region in formalin-preserved samples (3). The ITS-2 primers designed here to amplify a shorter region appear to allow identification through a smaller segment of the ITS-2. Snail samples were also tested but often yielded a mixture of ITS-2 amplicons from different trematode species (data not shown) and we don't recommend generic ITS-2 PCR without sequencing as a diagnostic method for this specimen type.

Whereas ITS-2 amplification and sequencing are relatively straightforward and routine, interpretation of sequence data to species level was challenging for several species due to inconsistencies in comparator data available in GenBank, particularly for *G. crumenifer*, which was distributed across three clades, with levels of intra-specific homology that were clear outliers relative to the distribution of pair-wise homologies within species (Figure 2). GenBank is a public data repository and is known to contain data errors (56). Only GenBank entries that matched closely with *C. cotylophorum* were classed as “unverified” but considering the difficulties in morphological identification of rumen fluke species, it is quite possible that genetic sequence data has been attributed without verification or incorrectly to other rumen fluke species too. To overcome this issue, development of an international, curated consensus reference database would be desirable. A Trematode.net database has been added to the veteran Nematode.net, as part of the collective Helminth.net, but as of yet, no paramphistome species are listed (57). We propose that ITS2 sequencing be used for definitive identification of paramphistomes. Where ITS2 comparator data is limited or lacking, as for *C. cotylophorum* in the current study, additional DNA sequencing could be used. For example, following a debate as to whether *P. cervi* and *P. leydeni* were distinct species, their complete mitochondrial genomes were compared and revealed a genetic divergence of 3.1% in the ITS-2 region, compared with a 14.7% variation in mtDNA (6). The higher divergence and resolving power of mtDNA also makes the Cox1 region preferable for examining genetic diversity within species (7, 21). Lack of published reference data made this method unsuitable for the heterogeneity of rumen fluke species considered in the current study.

Identification of *G. crumenifer* was particularly problematic. Only one specimen in our collection, originating from a water buffalo in India, belonged to this species, and it showed 100% genetic identity with one of the reported *G. crumenifer* sequences in GenBank. Identity with other reported *G. crumenifer* sequences, however, was low. Intraspecific variation has also been reported for the Cox1 region of *G. crumenifer* (16). It has been suggested that a high level of heterogeneity between isolates from different countries may suggest a higher degree of virulence (50) but in the absence of a gold standard, questions about species identity remain. The 28S rRNA region is being used increasingly for paramphistome species differentiation, due to its higher resolving power and greater nucleotide variation resolving previous issues with groupings of paramphistomes (46), but misclassification of original specimens could still affect interpretation of 28S data.

In conclusion, a total of 387 paramphistome specimens from 173 individual animals belonging to 10 host species and originating from 17 countries across multiple continents were successfully identified as 10 different paramphistome species through molecular methods. This demonstrates the utility of ITS2 sequencing as a generic approach for identification of paramphistomes, regardless of origin or phenotype. Moreover, the method is suitable for a variety of specimen types, including those with limited DNA template quality. Additional targets such as 28S (16) and Cox1 (51, 58) can be used to complement the available ITS-2 data, particularly to confirm identification of apparently new species. The lack of consistent clustering of rumen fluke species in the ITS2-phylogeny, combined with the fact that some supposed intra-specific ITS2 homologies fall far outside their normal distribution and well within the distribution of inter-specific homologies, raises concerns about the accuracy of attribution of sequence data to species in GenBank. Establishment of an international consensus reference database would be of great value to support accurate identification of rumen fluke species and studies of their distribution, emergence, virulence, and control.

## Data Availability Statement

The raw data supporting the conclusions of this article will be made available by the authors, without undue reservation.

## Author Contributions

PS and RZ conceived of the study. PS coordinated national and international collaborations and sample collection. GM developed the laboratory methodology and performed all analyses. GM and RZ drafted the manuscript. All authors reviewed the final manuscript.

## Conflict of Interest

The authors declare that the research was conducted in the absence of any commercial or financial relationships that could be construed as a potential conflict of interest.
