# Supplementary material for: A Universal Approach to Molecular Identification of Rumen Fluke Species Across Hosts, Continents, and Sample Types
Source: Front Vet Sci. 2021 Mar 4;7:605259. doi: 10.3389/fvets.2020.605259 (PMC7969503; doi:10.3389/fvets.2020.605259)
Supplement: Supplementary file 1 [file Data_Sheet_1.docx]

Supplementary Material

## Supplementary Figures

##
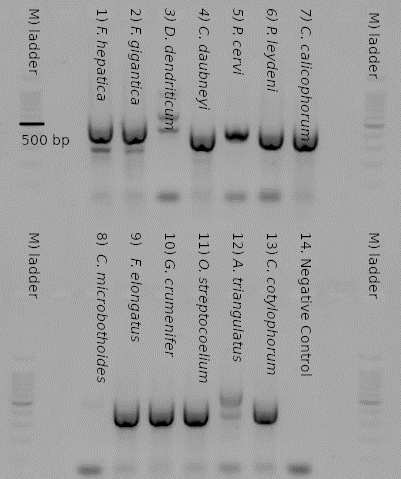


**Supplementary Figure 1.** PCR products amplified from 13 different trematode species using ITS-2 Trem primers. M - 100 bp ladder; 1 - *Fasciola hepatica* (515 bp); 2 - *Fasciola gigantica* (543 bp); 3 - *Dicrocoelium dendriticum* (540 bp); 4 - *Calicophoron daubneyi* (386 bp); 5 -*Paramphistomum cervi* (463 bp); 6 - *Paramphistomum leydeni* (417 bp); 7 - *Calicophoron calicophorum* (452 bp); 8 - *Calicophoron microbothrioides* (445 bp); 9 - *Fischoederius elongatus* (441 bp); 10 - *Gastrothylax crumenifer* (459 bp); 11 - *Orthocoelium streptocoelium* (448 bp); 12 - *Arthurdendyus triangulatus* (620 bp); 13 - *Cotylophoron cotylophorum* (467 bp); 14 - Negative control - NF H2O.

**Supplementary Figure 2.** Distribution of pair-wise sequence identity between all available ITS-2 GenBank (n=155) sequences and unique in-house generated sequences (n=13) for the 10 paramphistome species found in this study, including % identity below 90% (n = 174). Green bars represent interspecies comparisons (n = 11,926) and blue bars represent intraspecies comparisons (n = 2059) based on species names assigned in GenBank or through our study. Sequences were aligned using MEGA6 and trimmed to the same length of 299 bp before determining the number of base differences per site between sequences using the Pairwise Distance computation function. Values around 60% are mostly from comparison of one *Gastrothylax crumenifer* sequence (Accession number: KY889141) versus other sequences.


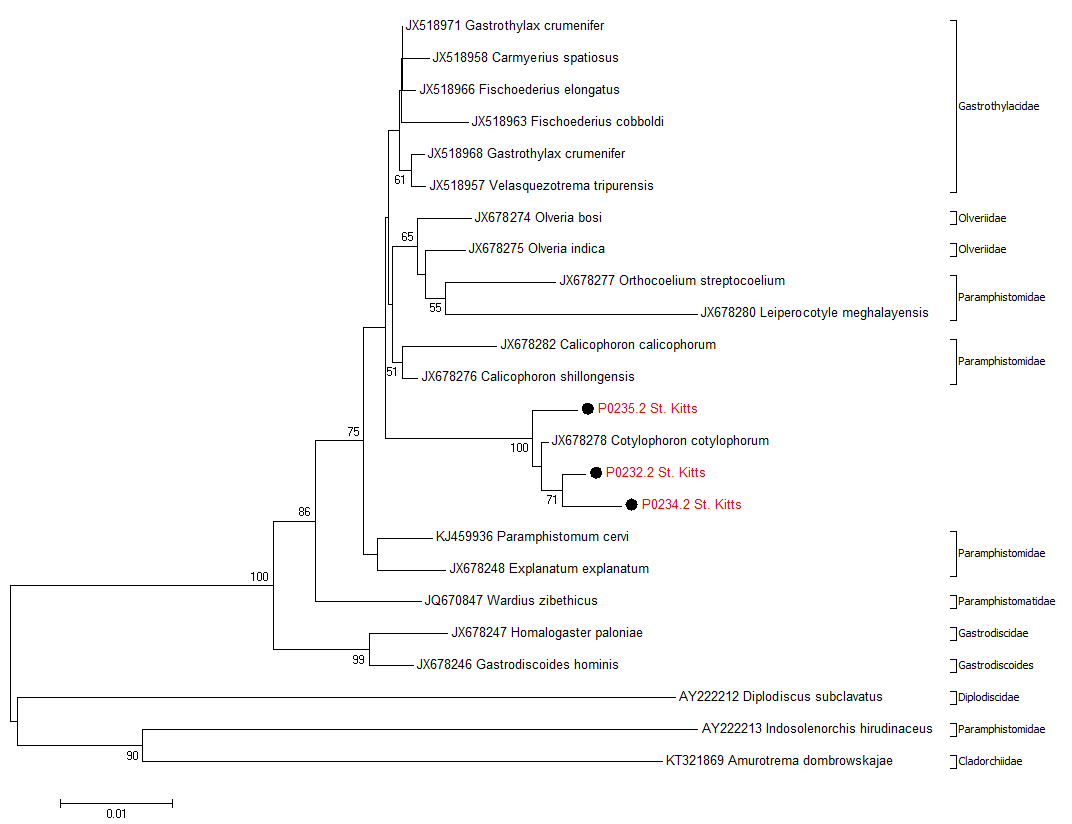


**Supplementary Figure 3:** Neighbour-joining tree showing 3 unique 28S rRNA sequences generated from St. Kitts paramphistomes in this study (P0232.2, P0234.2 and P0235.2) compared to those available on GenBank. The percentage of replicate trees in which the associated taxa clustered together in the bootstrap test (1000 replicates) is shown next to the branches. The evolutionary distances were computed using the Maximum Composite Likelihood method and are represented as number of base substitutions per site. Bootstrap values of <50% are not shown.

## Supplementary Tables

**Supplementary Table 2:** Statistics for the frequency distribution histogram (Figure 2 & S2) created for the pair-wise sequence comparison between all available ITS-2 GenBank (n=155) sequences and unique in-house generated sequences (n=13) for the 10 paramphistome species found in this study. Figure 2 and Figure S2 include 2046 versus 2059 and 11765 versus 11926 comparisons, respectively, whereby 174 comparisons with <90% homology were excluded from Figure 2.

|  | **Intraspecific** | **Interspecific** |
| --- | --- | --- |
| Pairwise comparisons |  |  |
| Total number | 2059 | 11926 |
| Number excluded ^1)^ | 13 | 161 |
| Number binned in histogram | 2046 | 11765 |
| Sequence homology (%) |  |  |
| Minimum | 60 | 57.91506 |
| 25% Percentile | 98.9011 | 96.337 |
| Median | 100 | 97.0696 |
| 75% Percentile | 100 | 97.8022 |
| 90% Percentile | 100 | 97.8 |
| 95% Percentile | 100 | 98.17 |
| Maximum | 100 | 100 |
| Mean | 98.97830248 | 96.47379466 |
| Std. Deviation | 3.314558823 | 4.35651805 |
| Std. Error of Mean | 0.073046188 | 0.039892579 |
| Lower 95% CI of mean | 98.83505033 | 96.39559871 |
| Upper 95% CI of mean | 99.12155463 | 96.55199062 |

^1)^ Comparisons were excluded if pairwise distance below 90% identity.
