# Supplementary material for: A Universal Approach to Molecular Identification of Rumen Fluke Species Across Hosts, Continents, and Sample Types
Source: Front Vet Sci. 2021 Mar 4;7:605259. doi: 10.3389/fvets.2020.605259 (PMC7969503; doi:10.3389/fvets.2020.605259)
Supplement: Supplementary file 2 [file Table_1.docx]

|  | **In-house** | **GenBank** | **TOTALS** |
| --- | --- | --- | --- |
| *Calicophoron calicophorum* | 2 | 3 | **5** |
| *Calicophoron daubneyi* | 1 | 17 | **18** |
| *Calicophoron microbothrioides* | 2 | 18 | **20** |
| *Calicophoron microbothrium* | 2 | 48 | **50** |
| *Cotylophoron cotylophoron* | 1 | 2 | **3** |
| *Fischoederius elongatus* | 1 | 13 | **14** |
| *Gastrothylax crumenifer* | 1 | 13 | **14** |
| *Orthocoelium streptocoelium* | 1 | 6 | **7** |
| *Paramphistomum cervi* | 1 | 12 | **13** |
| *Paramphistomum leydeni* | 1 | 23 | **24** |
| **TOTALS** | **13** | **155** | **168** |

**Supplementary Table 1:** Number of each species used in the distribution of pair-wise sequence identity analysis for the % identity histogram (Figure 2).
